# Thermal Cycling Behavior of Aged FeNiCoAlTiNb Cold-Rolled Shape Memory Alloys

**DOI:** 10.3390/mi15111338

**Published:** 2024-10-31

**Authors:** Li-Wei Tseng, Wei-Cheng Chen

**Affiliations:** Department of Mechatronics Engineering, National Changhua University of Education, Changhua 50007, Taiwan; d1151002@gm.ncue.edu.tw

**Keywords:** FeNiCoAlTiNb, shape memory property, texture intensity, recoverable strain, low-angle grain boundary

## Abstract

Fe–Ni–Co–Al-based systems have attracted a lot of interest due to their large recoverable strain. In this study, the microstructure and thermal cycling behaviors of Fe_41_Ni_28_Co_17_Al_11.5_Ti_1.25_Nb_1.25_ (at.%) 98.5% cold-rolled alloys after annealing treatment at 1277 °C for 1 h, followed by aging for 48 h at 600 °C, were investigated. From the electron backscatter diffraction results, we see that the texture intensity increased from 9.4 to 16.5 mud and the average grain size increased from 300 to 400 μm as the annealing time increased from 0.5 h to 1 h. The hardness results for different aging heat treatment conditions show the maximum value was reached for samples aged at 600 °C for 48 h (peak aging condition). The orientation distribution functions (ODFs) displayed by Goss, brass, and copper were the main textural features in the FeNiCoAlTiNb cold-rolled alloy. After annealing, strong Goss and brass textures were formed. The transmission electron microscopy (TEM) results show that the precipitate size was ~10 nm. The X-ray diffraction (XRD) results show a strong peak in the (111) and (200) planes of the austenite (⁠⁠γ, FCC) structure for the annealed sample. After aging, a new peak in the (111) plane of the precipitate (⁠⁠γ′, L12) structure emerged, and the peak intensity of austenite (⁠⁠γ, FCC) decreased. The magnetization–temperature curves of the aged sample show that both the magnetization and transformation temperature increased with the increasing magnetic fields. The shape memory properties show a fully recoverable strain of up to 2% at 400 MPa stress produced in the three-point bending test. However, the experimental recoverable strain values were lower than the theoretical values, possibly due to the fact that the volume fraction of the low-angle grain boundary (LABs) was small compared to the reported values (60%), and it was insufficient to suppress the beta phases. The beta phases made the grain boundaries brittle and deteriorated the ductility. On the fracture surface of samples after the three-point bending test, the fracture spread along the grain boundary, and the cross-section microstructural results show that the faces of the grain boundary were smooth, indicating that the grain boundary was brittle with an intergranular fracture.

## 1. Introduction

Shape memory alloys (SMAs) are smart materials with (a) shape memory effects (SMEs) and (b) superelasticity (SE) [1]. SMAs are widely used in different applications, such as actuators, wearable devices, robots, automotive and aerospace products, and biomedical devices [1,2]. SMAs can also be used in microdevices such as sensors and actuators [3]. In industrial applications, (1) wireless robots use SMAs as spring actuators in microrobots, (2) SMA can be used in radiofrequency identification antennas to detect temperature thresholds, (3) microdevices such as microrobots, microgrippers, and micropumps use SMAs as thermal actuators, and (4) SMAs can be used in gas and humidity sensors [3]. In biomedical engineering applications, (1) SMAs are used in microsurgery by temperature control actuators and (2) SMAs spring actuators are applied in medical gloves, which are used to restore the functionalities of a human hand [4].

Fe–Ni–Co–Al-based systems have attracted a lot of attention since 2010. Tanaka et al. [5] found that Fe_40.95_Ni_28_Co_17_Al_11.5_Ta_2.5_B_0.05_ (at.%) alloys show a large recoverable strain at room temperature. Fe–Ni–Co–Al–X (X = Ta, Ti, Nb) SMA systems have also been reported to have a greater than 2% recoverable strain [6,7,8,9,10,11,12,13,14,15,16,17]. In Fe–Ni–Co–Al-based systems, the austenite phase is face-centered cubic (fcc) and the martensite phase is body–centered tetragonal (bct) [5]. A strong recrystallizable texture can be achieved via cold-rolling and annealing heat treatment. Precipitates are obtained during the aging heat treatment, and their structure is L1_2_ [5].

The shape memory and superelasticity of <001>—oriented Fe_41_Ni_28_Co_17_Al_11.5_Ta_2.5_ (at.%) single crystals were first investigated by Ma et al. [18,19]. Based on their results, the optimal aging condition was taken as 600 °C for 90 hours (h). Under thermal 4545cycle testing, the tensile sample showed a 3.75 recoverable strain, and the compressive sample showed a 2% recoverable strain. The tensile sample displayed a 4% recoverable strain of superelasticity at 0 °C, and the compressive sample presented a 2% recoverable strain at 40 °C. The precipitate size and volume fraction were 3 nm and 38%, respectively. However, the recoverable strain levels were lower than the theoretical transformation strain values, possibly due to the large volume fraction of non-transforming precipitates, incomplete martensite reorientation, higher c/a ratio, and formation of TaC particles [19,20].

Chumlyakov et al. [21,22] found that adding Nb into an Fe–Ni–Co–Al-based system could improve the recoverable strain of superelasticity. Fe_41_Ni_28_Co_17_Al_11.5_Nb_2.5_ (at.%) <001>—oriented Fe_41_Ni_28_Co_17_Al_11.5_Nb_2.5_ (at.%) single crystals aged at 700 °C for 0.5 h showed a large recoverable strain of ~15.3% at −196 °C. This large recoverable strain was due to the formation of reversible mechanical twinning of the <011>{110} system. The diameter of the precipitate was less than 3 nm. The stress–temperature slope was 3.1 MPa/°C between −196 °C and −40 °C [23]. Furthermore, the effects of two-step aging heat treatment on the microstructure and superelasticity were reported in these studies [24]. The two-step aging conditions of FeNiCoAlNb single crystals with a <100> orientation were (a) 700 °C for 0.5 h and (b) 700 °C for 3 h. In the incremental strain test at −30 °C, the tensile sample showed a 7% superelastic strain and the compressive sample presented a 11.5% superelastic strain. In the 1% tensile and compressive strain test at various temperatures, the stress–temperature slope was 2.2 MPa/°C, from −196 °C to −25 °C. The irrecoverable compression was due to the retained martensite. The diameter of the precipitates was around 5 to 8 nm [25].

Tseng et al. [26] discovered that substituting Ti for Ta could decrease the aging time required to form precipitates. The aging condition of <001>-oriented Fe_41_Ni_28_Co_17_Al_11.5_Ti_2.5_ (at.%) single crystals was 600 °C for 4 h. In the incremental strain test at −80 °C, the tensile sample showed a 6% superelastic strain, and the compressive sample presented a 2% superelastic strain. The tension–compression asymmetry of <001>-oriented FeNiCoAlTi single crystals was due to variant selection. The constant strain test performed at different temperatures showed stress–temperature slopes of 3.3 MPa/°C under tension and 3.9 MPa/°C under compression, from −80 °C to room temperature. The precipitate size was around 5 nm. After increasing the aging time to 24 h, the tensile samples presented around 4.5% recoverable strain at room temperature. From the digital image correlation (DIC) results, we can see that one martensite variant was observed during the superelastic test. The magnetic results showed that the maximum magnetization was 160 emu/g under 7 Tesla [27]. In the TEM result, we see that the sizes of the precipitates were 10 nm and they possessed low Fe content and high Ni content.

FeNiCoAlTiB SMAs was first investigated by Lee et al. [7]. Based on their studies, the 98.5% cold-rolling sheet was annealed at 1200 °C for 3 h and then aged at 550 °C for 24 h, after which it presented a 4.2% recoverable strain. Later, Cassinerio et al. [28] studied the effects of different aging heat treatment conditions (600 °C, 650 °C, 700 °C) on the transformation temperatures of FeNiCoAlTiB wires. Different aging heat treatment conditions were found to affect the transformation temperatures due to the compositional changes in the austenite matrix. The composition of the precipitates included a Ni-rich phase, and the treatment reduced the Ni content in the austenite matrix. As a result, the transformation temperatures tended to increase with increasing aging times due to the depletion of Ni content in the austenitic matrix [28,29,30].

Omori et al. [6] studied the microstructure and superelasticity of FeNiCoAlNbB SMAs under tension testing. The 98.5% cold-rolling sheet was annealed at 1220 °C for 1 h and then aged at 600 °C for 96 h, presenting a 5% recoverable strain. The recrystallization textures in RD were in the <110> orientation. Later, Fu et al. [31] investigated the effects of cold-rolling on the texture and superelastic properties. With cold-rolling reduction below 80%, a weak copper texture appeared. With cold-rolling reduction up to 98.5%, strong Goss and brass textures were formed in the sample. The aged sample (600 °C, 96 h) showed a 3.2% recoverable strain, and the volume fraction of the low-angle grain boundary was 53%.

Although adding boron into an Fe–Ni–Co–Al-based system can suppress the beta phases generated during the aging heat treatment, it extends the aging times required to form precipitates [32]. Moreover, it is difficult to precisely control the small amount of boron added into alloys during arch melting [30]. As a result, we added Nb to strengthen the matrix, and added Ti to reduce the aging time in Fe–Ni–Co–Al-based system. In this study, Fe_41_Ni_28_Co_17_Al_11.5_Ti_12.5_Nb_12.5_ (at.%) cold-rolled alloys aged at 600 °C for 48 h were investigated for their microstructure, crystal structure, magnetic properties, and shape memory properties under a three-point bending test.

## 2. Materials and Methods

An Fe_41_Ni_28_Co_17_Al_11.5_Ti_1.25_Nb_1.25_ (at.%) ingot was fabricated by vacuum induction melting (VIM). Wire electrical discharge machining (EDM) was used to cut an ingot into several blocks. Each block with the dimensions of 25 × 25 × 100 mm (thickness, width, and length) was solution-treated at 1277 °C for 24 h and then directly cold-rolled at 98.5% (98.5%CR) to 0.375 mm. These specimens were annealed and heat-treated at 1277 °C for 1 h, followed by water quenching, and then aged at 600 °C for 48 h. Electron backscatter diffraction (EBSD) was performed to investigate the texture, grain size, and misorientation of the sample after annealed heat treatment. The surface quality of EBSD samples were finally polished to 0.05 μm using diamond suspension (0.05 μm). The etching solution used in preparing the sample’s surface was 90% C_2_H_5_OH + 10% HClO_4_. The SEM images of the fractured surface were obtained using a JeoL JSM-7800F device (JEOL, Musashino, Akishima, Japan). The voltage was 20 V. The BSE images were characterized by Scanning Electron Microscopy (SEM). The magnetization–temperature (M–T) curves of the aged samples were characterized using a Superconducting Quantum Interference Device (SQUID) in magnetic fields of 0.05, 1, 3, 5, and 7 Tesla. The SQUID device was MPMS-3 (Quantum Design, San Diego, CA, USA). The hardness of the aged specimens was measured using a Vickers hardness device with an FM-310 (FUTURE-TECH CORP, Kawasaki, Japan). The microstructures of the aged samples were observed using an Olympus digital optical microscope. The etching solution was composed of 7% nitric acid and 93% ethanol.

The precipitate size in the aged sample was characterized with transmission electron microscopy (TEM). A focused ion beam (FIB) was used to prepare the TEM sample. The bright-field TEM image and the corresponding selected-area electron diffraction pattern were characterized using a JEOL JEM-F200 electron microscope. The crystal structures of samples were analyzed by X-ray diffraction (XRD). The model used was a D5000 X-ray diffraction device (Siemens, Aubrey, TX, USA). The 2 Thea range is from 30 to 100 degrees. The samples, 98.5%CR and 98.5%CR after annealing and heat treatment at 1277 °C for 1 h (98.5%CR + 1277 °C–1 h), were assessed by XRD. The calculations of the orientation distribution functions (ODFs) employed the pole figure results. The planes of the pole figures were selected as (111), (200), and (220). The textural components of the FCC structure were phi 2 = 0°, 45°, and 65°sections of the Euler space.

The thermal cyclic behavior of the aged FeNiCoAlTiNb sample was determined via a three-point bending test with a ElectroForce 3230 (TA Instruments, New Castle, DE, USA). The support span was 20 mm. The heating/cooling cyclic temperature range was between 50 °C and −150 °C. The shape memory responses were observed under different applied stress levels: 100, 200, 300, and 400 MPa. The sample broke as the stress level increased to 500 MPa.

## 3. Results and Discussion

### 3.1. EBSD and ODF Results

**Figure 1:**
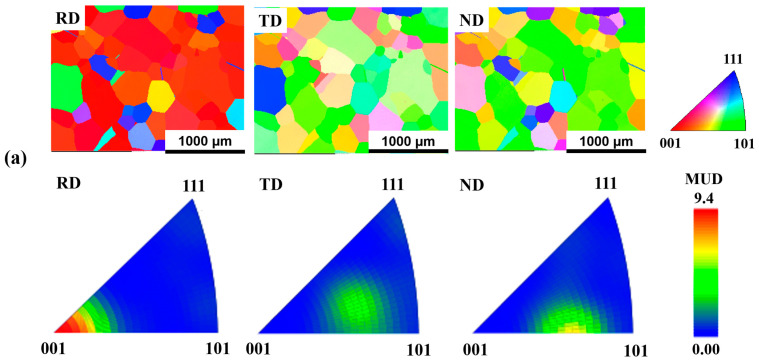

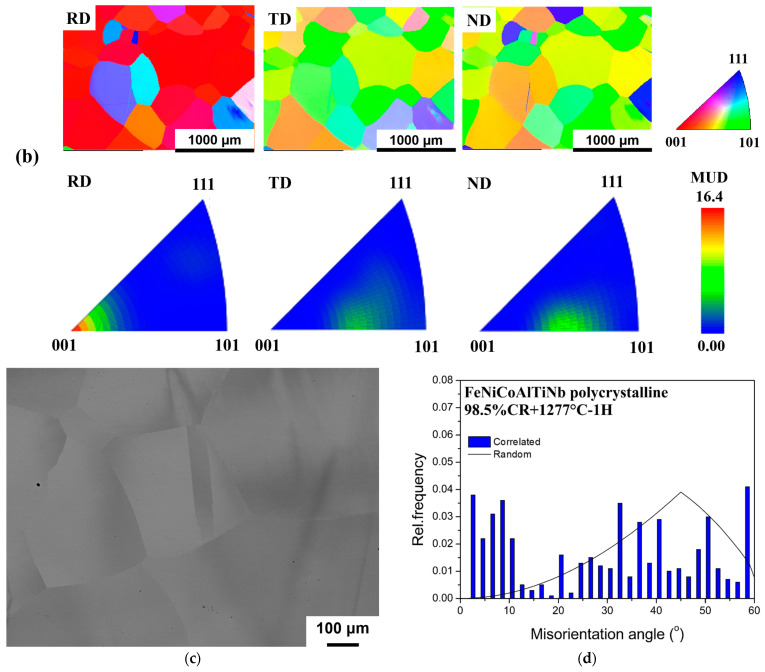
EBSD pattern of the FeNiCoAlTiNb cold-rolled alloys annealed at 1277 °C for (**a**) 0.5 h and (**b**) 1 h. (**c**) BSE image and (**d**) grain boundary misorientation of the 98.5%CR + 1277 °C, 1 h sample. RD (rolling direction), TD (transverse direction), and ND (normal direction).

Figure 1a,b present the EBSD inverse pole figures (IPFs) for FeNiCoAlTiNb cold-rolled alloys under different annealing conditions: 98.5%CR + 1277 °C, 0.5 h and 98.5%CR + 1277 °C, 1 h. In IPFs, the abbreviations were RD for the rolling direction, TD for the transverse direction, and ND for the normal direction. From the EBSD results, we can see that the texture intensity of the RD was in the <100> orientation. For the 98.5%CR + 1277 °C, 0.5 h sample, the maximum textural intensity was 9.4 mud. For the 98.5%CR + 1277 °C, 1 h sample, the maximum textural intensity was 16.4 mud. The average grain size was approximately 300 to 400 μm for 98.5%CR + 1277 °C, 0.5 h and 98.5%CR + 1277 °C, 1 h, respectively. Based on the maximum textural intensity and average grain size results, the annealed heat treatment times were set as one hour in this study. Figure 1c shows a BSE image of the 98.5%CR + 1277 °C, 1 h sample. The grain boundary is clear, without forming the beta phases before the aging heat treatment was applied. Figure 1d shows the grain boundary misorientation of the 98.5%CR + 1277 °C, 1 h sample. The fraction of LABs was about 15.7%. The fractions of LABs in the present alloys are slightly smaller compared with those of the FeNiCoAlNbB alloy [31], and much smaller than those of the FeNiCoAlTaB alloy (60%) [5].

**Figure 2:**
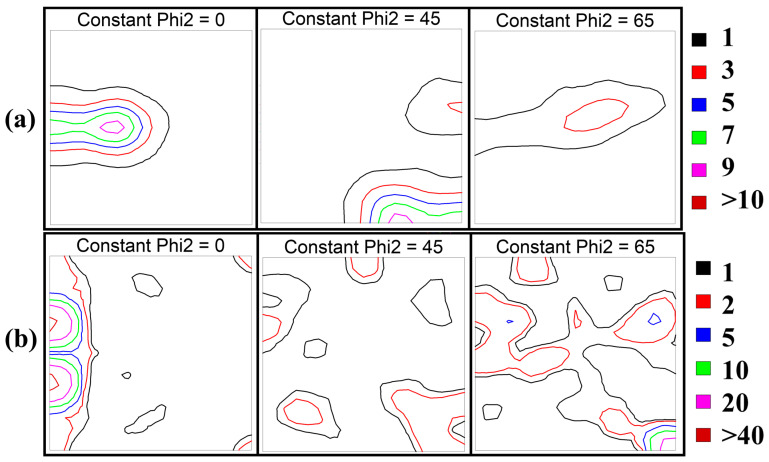
ODFs of the FeNiCoAlTiNb cold-rolled alloys: (**a**) 98.5%CR and (**b**) 98.5%CR + 1277 °C, 1 h.

Figure 2a,b show the ODFs (phi 2 = 0°, 45°, and 65° sections) for 98.5%CR and 98.5%CR + 1277 °C, 1 h. Copper ({112} 〈111〉), brass ({110}〈112〉), and Goss ({110}〈001〉) textures are the three major components in FeNiCoAlTiNb cold-rolling alloys [31]. The 98.5%CR sample showed a strong rolling texture in terms of both brass ({110}〈112〉) and Goss ({110}〈001〉). It showed a weak copper texture. f(g) is the orientation density and is commonly used in the cold-rolling textures of FCC metals. f(g)_max_ represents the maximum value of orientation density. The textural intensity was f(g)_max_ = 10.79 for brass texture, f(g)_max_ = 9.07 for Goss texture, and f(g)_max_ = 4.14 for copper texture. The cold-rolled sample after annealing showed a texture intensity of f(g)_max_ = 45.14 for the brass texture and f(g)_max_ = 46.14 for the Goss texture. The textural intensities of both the Goss and brass textures increased, and the intensity of the copper texture decreased. Furthermore, the f(g)_max_ of recrystallization texture reached 46.14. From the ODFs results, we see that the two major components of FeNiCoAlTiNb 98.5%CR + 1277 °C, 1 h were brass ({110}〈112〉) and Goss ({110}〈001〉) texture. Moreover, many researchers have shown that cold-rolling above 98% forms {hkl}<100> recrystallization textures.

### 3.2. Hardness Result

**Figure 3:**
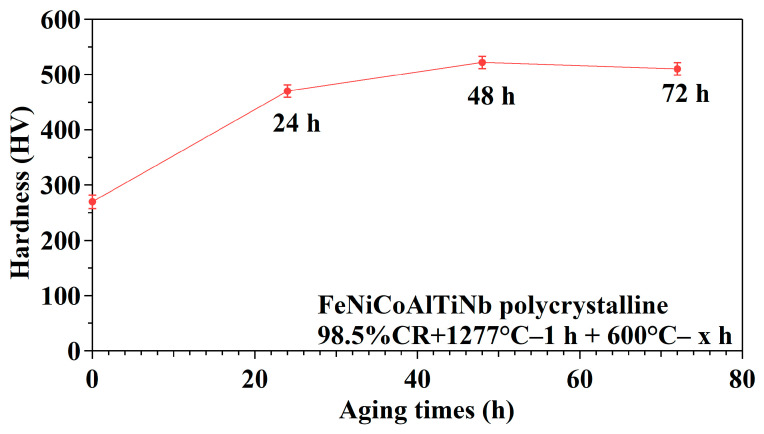
Hardness of a FeNiCoAlTiNb 98.5%CR with a different aging treatment duration.

Figure 3 presents room temperature hardness test results. The hardness values produced with 24, 48, and 72 h of aging are 470, 522, and 510 HV, respectively. The hardness value increased from 24 to 48 h. As the aging time reached 48 h, the hardness value began to decrease. As a result, the aging time of 48 h was selected as the peak aging condition for the further microstructure, XRD, SQUID, TEM, and three-point bending tests.

### 3.3. XRD Results

**Figure 4:**
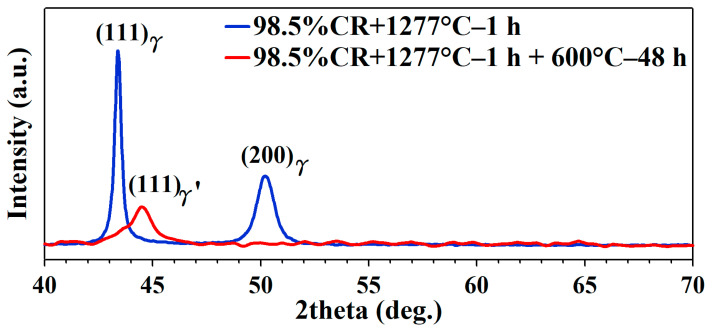
XRD pattern for FeNiCoAlTiNb cold-rolled alloys under different thermomechanical processing times.

Figure 4 presents the XRD patterns of the samples under different conditions, including 98.5%CR + 1277 °C, 1 h and 98.5%CR + 1277 °C, 1 h + 600 °C, 48 h. In the annealing sample, we see a strong peak in the (111) and (200) planes for the austenite (⁠⁠γ, FCC) structure. The intensity of the (111) peak was higher compared to the (200) peak. In the aged samples, a new peak was generated, and the two theta values reached between 44 and 45 degrees. In the reference results, this peak was identified in precipitates, and its plane was (111), contributing (⁠⁠γ′, L12). The original (111) and (200) plane peaks of the annealing sample decreased after the aging heat treatment. Similar phenomena were also reported by Zhou et al. [33]. In their studies, the peak intensity of austenite (⁠⁠γ, FCC) decreased and the peak intensity of precipitate (⁠⁠γ′, L12) increased with the aging times 24 to 96 h. Beta phases appeared between 64 and 65 degrees. For our sample, the peak of the beta phase was unclear compared to that of Fu et al. [31].

### 3.4. TEM Results at Room Temperature

**Figure 5:**
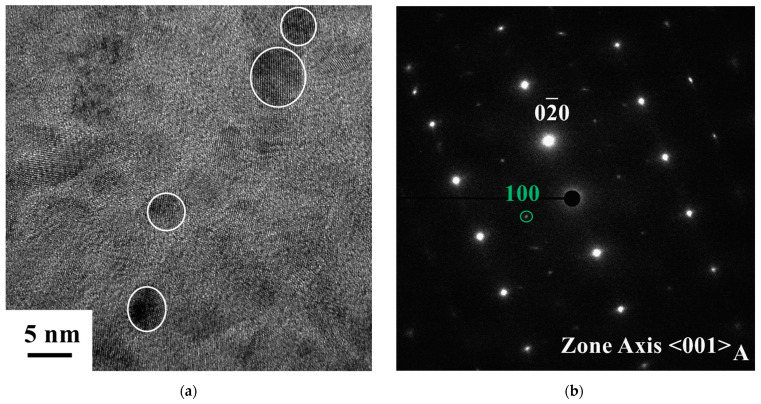
TEM images of the aged FeNiCoAlTiNb sample: (**a**) BF TEM image and (**b**) SAED pattern. The white circle shows the size of the precipitate.

Figure 5a shows the bright-field (BF) TEM image of an aged FeNiCoAlTiNb sample. The white circle shows the diameter of the precipitate. The precipitate size was measured as 10 nm. The SAED patterns of the aged sample are shown in Figure 5b. The axial zone is <001>. Based on the diffraction pattern results, we can infer that the austenite phase has an fcc structure and the precipitate has an L1_2_ structure. In the results for the FeNiCoAlNb single crystal with a <100> orientation, the diameter of the 700 °C, 3 h aged FeNiCoAlNb single crystal was around 5 nm [21]. For the FeNiCoAlTiB alloy aged at 600 °C for 4 h and 600 °C for 24 h, the precipitate sizes were 4 and 10 nm, respectively [26,27].

### 3.5. Magnetization Results of FeNiCoAlTiNb Aged Sample

**Figure 6:**
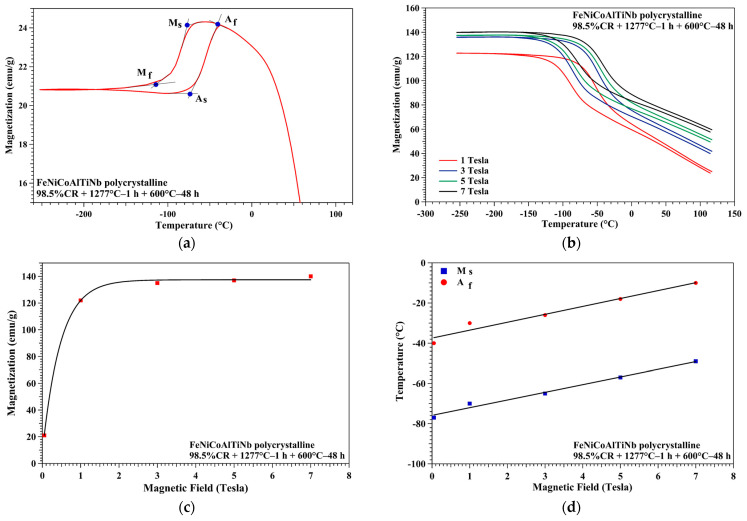
Magnetization vs. temperature at various levels of magnetic field for the aged FeNiCoAlTiNb sample: (**a**) 0.05 T, (**b**) 1, 3, 5, and 7 T. (**c**) Magnetic field vs. magnetization response and (**d**) magnetic field vs. temperature response.

Figure 6a,b present the magnetic results for the FeNiCoAlTiNb sample aged under magnetic fields of 0.05, 1, 3, 5, and 7 Tesla. The transformation temperatures of the aged sample were determined by the tangent line method, as shown in Figure 6a. The values of the transformation temperatures and temperature hysteresis under different magnetic fields are summarized in Table 1. The austenite finish temperature (A_f_) here = −40 °C and the martensite start temperature (M_s_) = −77 °C. The temperature hysteresis (ΔT) was calculated as |A_f_ − M_s_|, and its value ranged from 37 to 39 °C for the magnetic fields of 0.05, 1, 3, 5, and 7 Tesla. Figure 6b shows that magnetization increases with increases in the magnetic field from 1 to 7 Tesla. Figure 6c shows the relationship between magnetization and the magnetic field. The magnetization increased with an increasing magnetic field. When the magnetic field was 5 T, the magnetization almost reached its saturation value. The maximum value of magnetization was 140 emu/g at 7 Tesla. The results indicate that the magnetization increased with an increasing magnetic field. Figure 6d presents transformation temperatures (A_f_ and M_s_) as a function of the magnetic field at various values (0.05~7 Tesla). From this result, we can infer that the transformation temperature increases as the magnetic field increases.

### 3.6. Thermal Cyclic Results

**Figure 7:**
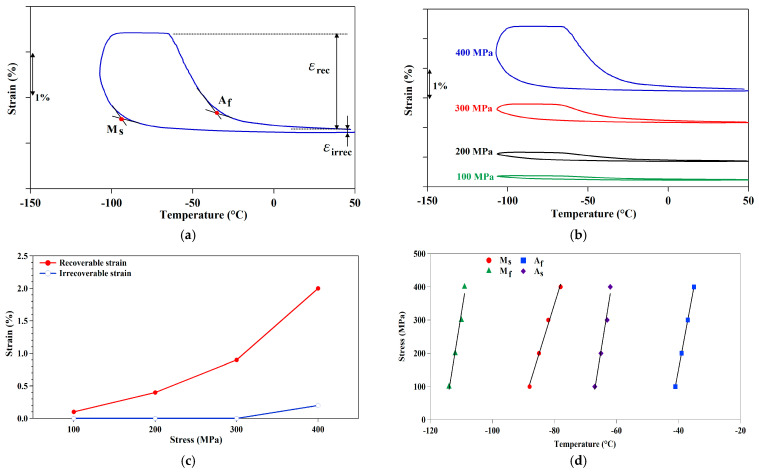
Shape memory characteristics of three-point bending test for the aged FeNiCoAlTiNb sample: (**a**) illustration of how to obtain recoverable strain, irrecoverable strain, and transformation temperatures from thermal cyclic tests, (**b**) strain vs. temperatures under different applied stress values, (**c**) recoverable and irrecoverable strains at various stress, and (**d**) stress vs. transformation temperatures responses.

Shape memory properties, such as recoverable strain, irrecoverable strain, and transformation temperatures, were determined as a function of applied stress in the thermal cyclic test results, as shown in Figure 7a. Figure 7b presents the thermal cycling result of the 98.5%CR + 1277 °C, 1 h + 600 °C, 48 h sample. The samples fractured under 500 MPa. The recoverable strain and irrecoverable strain as a function of applied stress were summarized in Figure 7c. The maximum recoverable strain was achieved at approximately 2%. The relationship between applied stress and transformation temperatures is summarized in Figure 7d. From the results, we can see that the transformation temperatures slightly increased when the stress levels increased from 100 MPa to 400 MPa. The stress–temperature slopes were 30 MPa/°C and 33 MPa/°C for the martensite start temperature and austenite start temperature, respectively. The stress–temperature curves in SMAs can be described by the Clausius–Clapeyron relationship,

(1) $$\frac{d\sigma }{dT}=-\frac{\Delta H}{\varepsilon_{tr}.T_{0}}$$

where dσ/dT is the stress–temperature slope; ΔH is the transformation enthalpy; ε_tr_ is the recoverable or transformation strain; and T_0_ is the phase equilibrium temperature. The stress–temperature slope is inversely proportional to the recoverable strain. The small recoverable strain indicates that the samples present large values of the stress–temperature slope, implying a lower recoverable strain.

**Figure 8:**
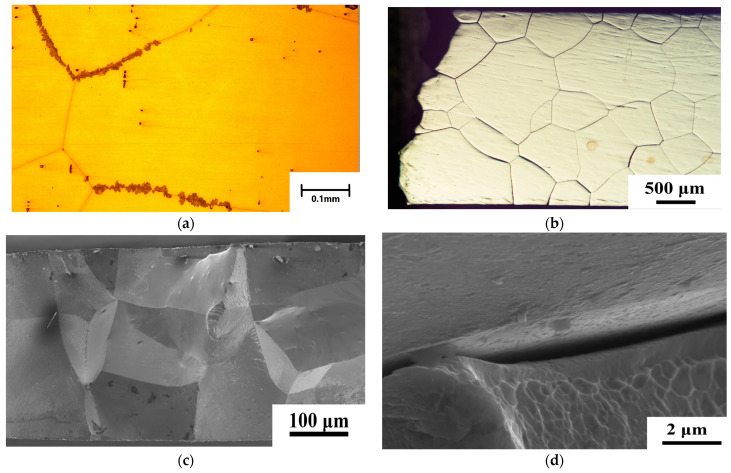
An optical microscope image of an aged sample: (**a**) before and (**b**) after the three-point bending test. (**c**,**d**) show high-magnification BSE images of the cross-sections of three-point bending fracture surfaces.

Based on the results of the shape memory bending test, the recoverable strain of the aged sample was 2%, which is smaller than the recoverable strain for FeNiCoAlTaB SMAs [3]. There are two possible reasons for this. First, the volume fraction of LABs was 15.7%, which is still lower than the LABs of 60% reported by Tanaka et al. [5]. In the Fe–Ni–Co–Al-based system, beta phases are generated during the aging heat treatment. These phases prefer to accumulate at the grain boundary. Beta phases cause the recoverable strain to deteriorate and form at high-angle grain boundaries (HABs). The development of LABs is important for suppressing the precipitation of β–NiAl at the grain boundaries. For the FeNiCoAlTiNb (98.5%CR + 1277 °C, 1 h + 600 °C, 48 h) aged sample, the optimal microstructure shows beta phase accumulation in the triple junction, as shown in Figure 8a. Figure 8b shows the fracture surface of the FeNiCoAlTiNb (98.5%CR + 1277 °C, 1 h + 600 °C, 48 h) aged sample after the three-point bending test. From the result, we see that the fracture developed along the grain boundaries. Figure 8c,d show higher-magnification BSE images of the cross-sections of the three-point bending fracture surfaces in the aged FeNiCoAlTiNb sample. The BSE images show that the faces of the grain boundary facets were smooth. The results indicate that the grain boundary is brittle and features an intergranular fracture. The same observation was reported by Zhang et al. [11]. Based on their results, when the FeNiCoAlTaB CR97.2 cold-rolled alloy was aged at 600 °C for 48 h, beta phases were generated along the grain boundary and affected the fracture mode and ductility of the FeNiCoAlTaB sample. The grain boundary fracture surfaces were smooth, indicating intergranular fracturing [11].

Secondly, the texture intensity in the <100> orientation was 16.4 in this study, which is still smaller than the texture intensity (25.4) of FeNiCoAlTaB SMAs [5]. Grain orientation is important in iron-based SMAs, and affect the shape memory properties. In an Fe–Ni–Co–Al-based system, there are three martensite variants that arise during fcc to bct transformation [20,21]. The maximum theoretical transformation strain in tension is 8.7% in the <100> direction, and the minimum theoretical transformation strain is 2.1% in the <111> direction [5]. The elastic anisotropy leads to incompatibilities between neighboring grains, and grain constraint becomes intense. A large number of constrained grains causes incompatibility in the transformation strain at grain boundaries. The martensitic transformation is suppressed, and fracturing will occur at the grain boundary. As a result, this directly limits the ability of FeNiCoAlTiNb samples to obtain large recoverable strain.

## 4. Conclusions

In summary, the microstructure, hardness, thermo-magnetization, and thermal cyclic behavior of the aged FeNiCoAlTiNb 98.5%CR sample were investigated in this study. The following conclusions can be made:

(1) The EBSD results reveal that the texture intensity and grain size increased as the annealing time increased from 0.5 to 1 h. After annealing at 1277 °C for 1 h, FeNiCoAlTiNb 98.5%CR showed a strong texture intensity in the <100> orientation, the value of which was 16.4 mud, and the average grain size was 400 μm. The volume fraction of LABs was 15.7%. The ODF results show the Goss texture and brass texture of the 98.5%CR sample after annealing;
(2) The hardness results show that the hardness value increased when the aging time increased from 24 to 48 h. After 48 h, the hardness value decreased. This result indicates that the aging condition employed here (600 °C, 48 h) is optimal;
(3) The TEM results indicate that the precipitate size in aged samples was around 10 nm. The crystal structure of precipitates was L1_2_. The XRD results reveal that the (111) and (200) plane peaks had an austenite (⁠⁠γ, FCC) structure in the annealing sample. After aging, the original (111) and (200) plane peaks decreased, and the (111) plane of the new peak increased, indicating precipitate (⁠⁠γ’, L1_2_);
(4) Based on the magnetic results, the magnetization of the aged samples increased with increases in the magnetic field. When the magnetic field approached 5 T, the magnetization almost reached its saturation value. The maximum magnetization was around 140 emu/g. As the magnetic field levels increased from 0.05 to 7 T, the transformation temperatures increased. This property (magnetic induction of the martensitic transformation) can be used for designing actuators where the elements of microrobot elongate and contract in response to changes in the magnetic fields.
(5) The thermal cyclic behavior of the aged sample showed a recoverable strain of 2% at the 400 MPa stress level during the three-point bending test. The observed recoverable strain values were lower than the theoretical values, possibly owing to the generation of grain boundary precipitates affecting the fracture mode and ductility of the aged sample, and this limits the sample’s ability to achieve a higher recoverable strain.
(6) The new FeNiCoAlTiNb SMAs have great potential applications in microdevices, such as actuation (actuator of micropump, gripper, and robot), temperature sensing (gas and humidity sensor), and thermal energy harvesting (generators).

## Figures and Tables

**Table 1:** Thermomagnetic properties of the aged FeNiCoAlTiNb sample under different magnetic fields.

Magnetic Field	Transformation Temperature (°C)	Temperature Hysteresis (°C)
0.05 T	A_f_ = −40 and M_s_ = −77	37
1 T	A_f_ = −30 and M_s_ = −68	38
3 T	A_f_ = −26 and M_s_ = −65	39
5 T	A_f_ = −18 and M_s_ = −57	39
7T	A_f_ = −10 and M_s_ = −49	39

## Data Availability

The original contributions presented in the study are included in the article, further inquiries can be directed to the corresponding author.
